# Transglycosylation of Stevioside by a Commercial β-Glucanase with Fungal Extracted β-Glucans as Donors

**DOI:** 10.1007/s12649-023-02052-4

**Published:** 2023-01-24

**Authors:** Anastasia Zerva, Milad Mohammadi, Georgios Dimopoulos, Petros Taoukis, Evangelos Topakas

**Affiliations:** 1 Biotechnology Laboratory, School of Chemical Engineering, National Technical University of Athens, 5 Iroon Polytechniou Str., Zografou Campus, 15780 Athens, Greece; 2 Laboratory of Food Chemistry and Technology, School of Chemical Engineering, National Technical University of Athens, 5 Iroon Polytechniou Str., Zografou Campus, 15780 Athens, Greece

**Keywords:** Stevioside, Transglycosylation, β-Glucanase, β-Glucan valorization, *Pleurotus*

## Abstract

**Abstract:**

Alternative sweeteners, such as steviol glucosides from the plant *Stevia rebaudiana* Bertoni, are becoming increasingly popular for the design of next-generation foodstuffs. However, the bitter aftertaste of native steviol glucosides is one of the main reasons behind consumer reluctance towards stevia-containing products. Biocatalysis could be a sustainable solution to this problem, through addition of glucosyl moieties to the molecule. Glycoside hydrolases are enzymes performing transglycosylation reactions, and they can be exploited for such modifications. In the present work, the commercial β-glucanase Finizym 250L® was employed for the transglycosylation of stevioside. After optimization of several reaction parameters, the maximal reaction yield obtained was 19%, with barley β-glucan as the glycosyl donor. With the aim to develop a sustainable process, β-glucan extracts from different fungal sources were prepared. Pulsed Electric Field pretreatment of mycelial biomass resulted in extracts with higher β-glucan content. The extracts were tested as alternative glucosyl donors, reaching up to 15.5% conversion yield, from *Pleurotus*-extracted β-glucan. Overall, in the present work a novel enzymatic process for the modification of stevioside is proposed, with concomitant valorization of β-glucans extracted from fungal biomass, potentially generated as a byproduct from other applications, in concert with the principles of circular economy.

**Graphical Abstract:**

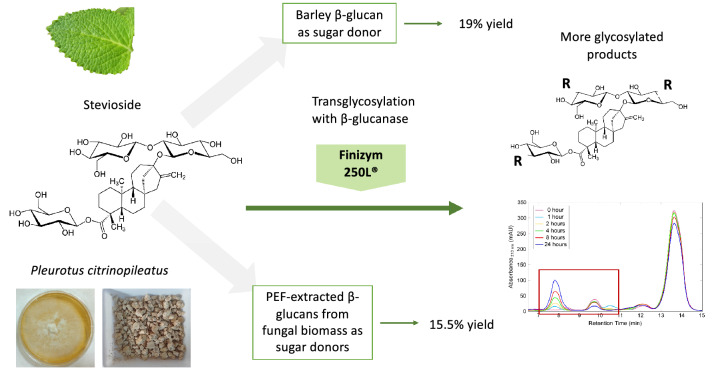

## Statement of Novelty

The transglycosylation of stevia glucosides could contribute to their wider consumer acceptance, due to the removal of bitter aftertaste. In the present work, a novel process is described for the transglycosylation of stevioside, with the use of a food-grade β-glucanase enzyme and spent mushroom biomass as source of β-glucans. Pulsed Electric Field pretreatment, a novel method, was employed for the extraction of β-glucan from the mushroom biomass, leading to higher yields of the transglycosylation reaction. Overall, the results of the present work could be used for the design of a cost-effective, green process for the sweetness improvement of stevia-containing products.

## Introduction

Steviol glucosides, extracted from the *Stevia rebaudiana* Bertoni native American plant, are one of the most popular sweeteners nowadays, due to their strong sweet taste and natural origin. However, the bitter aftertaste of natural steviol glucosides is an important drawback, leading to consumer reluctance. Stevioside and rebaudioside A (RebA), the most abundant steviosides in stevia leaves, showed significant bitterness in consumer-based sensory studies [1], while the more heavily glycosylated RebD and M, showed a sweetness profile similar to sucrose. A homology modelling study of stevioside and RebA with the human bitter taste receptor hTAS2R4 revealed that the extra glucosyl unit of RebA restricts the access to the receptor, and therefore the taste of RebA is perceived as sweeter than that of stevioside [2]. Generally, the addition of extra sugar moieties to the stevioside molecule has been shown to improve its taste profile, by a number of studies [1–6]. However, the addition of more than 1–2 glucosyl units can decrease sweetness, as shown by a recent study [3]. As shown by Wang et al. [3], the modification of a mixture of steviol glucosides by cyclodextrin glucanotransferase (CTGase) and α-glucosidase led to the reduction of the bitter aftertaste and the liquorice flavour, while the final products presented a unique flavour, different from the glucosides naturally present in stevia extracts. The authors also showed that increase in the glycosyl chain length, above a certain extent, decreased the sweetness of the final product, and thus the addition of an α-glucosidase to the reaction was necessary, to trim CTGase products, finally yielding steviosides with 1–2 extra glucose units [3].

Although the use of enzymes, such as CGTase, introducing α-glycosidic bonds to steviosides, has been applied successfully, this is rather undesirable for the final product, due to the activity of amylases in the human saliva, which are able to cleave those bonds and release glucose molecules, ultimately increasing the caloric value of the product [7]. In this respect, β-glucosidic bonds are favourable for the design of products for diabetic or obese consumers. Significant efforts have been devoted to the transglycosylation of steviosides with UDP-glucosyltransferases, the native stevia enzymes and their microbial homologues. Their requirement of UDP-activated donors has been circumvented with the co-expression of enzymes, such as sucrose synthase, regenerating the UDP donors [8–11].

Aside from their applications as sweeteners, steviol glucosides also possess very interesting bioactivities. Stevioside was recently found to induce apoptosis in bladder cancer cells in vitro and inhibited their growth in vivo [12]. Steviol glucosides have also been shown to possess prebiotic effects [13].

For all these reasons, the demand for steviol glucosides has been increasing in the last few years, leading to intensive research efforts for the cost-effective production of steviol sweeteners, eliminating the need for plant extraction. Modern approaches focus on the reconstruction of the biosynthetic path of steviol production on engineered microbes, such as the yeast *Saccharomyces cerevisiae* [14] or the bacterium *Escherichia coli* [15]. Such efforts lead to increased yields of steviol and other compounds that can be used as scaffolds for the production of novel steviosides with improved properties.

Previous efforts towards the production of unconventional steviol glucosides include the use of glycoside hydrolases [16, 17] for the introduction of alternative sugars in the stevioside backbone, as candidates for new bioactive compounds. Glycoside hydrolases are represented in microbial genomes from genes coding for a variety of enzymes with different specificities including enzymes for the degradation of β-1,3, β-1,6, β-1,3/1,4 β-1,3/1,6 glycosidic bonds in polymeric substrates. β-Glucanases, together with microbial cell wall β-glucans, play a major role in host–pathogen interactions in many pathogenic species, to the extent that β-glucanases have been used as biosensors for the detection of pathogens [18]. β-1,6-Glucanases are produced by bacteria growing on fungal cell walls as carbon source [19], but they are also present in fungi [20].

In fungi, β-glucanases participate in the formation of cell wall, but also in the degradation of plant biomass polysaccharides of variable structure. For example, β-1,3/1,4 glucanases are able to degrade lichenan, but also β-1,3/1,4 glucan from barley [21, 22], releasing oligosaccharides acting as carbon source for the organism, while β-1,3/1,6 glucanases are considered to participate in the synthesis and degradation of the fungal cell wall under starvation conditions [23]. Recently, new promiscuous β-glucanases have been discovered, with the ability to degrade all possible β-glucosidic bonds [24].

Most β-glucanases described so far also have transglycosylating activities, similarly to most glycoside hydrolases. Transglycosylating activity has been described for β-1,3–1,6-glucanases [25], β-1,3/1,4-glucanases [26, 27], β-1,3-glucanases [28] and others. So far, the production of laminarioligosaccharides [25] and mixed-linkage β-1,3 and β-1,4 oligosaccharides [26, 27], has been described, but also the grafting of xyloglucan oligosaccharides to cellulose [29]. However, there is only one previous report in the literature regarding the transglycosylation of stevioside by a β-glucanase from the white-rot fungus *Irpex lacteus* [2]. Therefore, with the use of β-glucanases, β-glucans from spent fungal biomass generated as a byproduct from other applications could be used as a raw material for stevioside transglycosylation. This could enable the design of bioprocesses in line with the Biorefinery concept and the principles of circular bioeconomy.

β-Glucanases are enzymes with great biotechnological interest. One of their most prominent applications is in the brewing industry, where they are used for the degradation of barley β-glucans, lowering the viscosity and turbidity of the mash [21]. Novozyme’s Finizym 250L® is a food grade, thermostable enzyme preparation, designed for this purpose. Moreover, β-glucanases can be used in detergents, for the removal of β-glucan-containing, food-related stains, in animal feed production, to increase the digestibility of the feed, but also for the production of prebiotic oligosaccharides [21].

In the present study, the commercial β-glucanase-containing enzyme preparation Finizym 250L® was used for the modification of stevioside. β-glucans from various sources were used as sugar donors, aiming at the removal of bitter aftertaste from stevioside. This is the first report for the use of fungal β-glucans as donors for stevioside transglycosylation, resulting in a sustainable and circular process.

## Materials and Methods

### Enzymes and Chemicals

Finizym 250L® was supplied from Novozymes A/S. Barley β-glucan (medium viscosity) was purchased from Megazyme (Bray, Co. Wicklow, Ireland). Stevioside (> 85%) was purchased from TCI America (Boston, MA, USA). All other chemicals were purchased from Merck KGaA, Darmstadt, Germany, and they were of the highest purity available.

### Production of β-Glucan Extracts from Microbial Sources

For the production of β-glucan from *S. cerevisiae,* commercially available instant dry yeast was used as starting biomass (*S. cerevisiae*, Angel Yeast Co. Ltd., Hubei, China, Lot no. 201811 CG). Instant yeast pellets were reconstituted in warm deionized water (30 °C) under gentle stirring for 20 min, in the form of a suspension with a concentration of 10% (w/w). After plating on YPD-Agar medium, it was determined that the viable count of the cells in the suspension after reconstitution was 9.2 logCFU mL^−1^. Following reconstitution, the suspension was centrifuged at 10,000×*g* for 10 min, and the resulting pellet was washed three times with equal volumes of deionized water. The pellet was finally freeze-dried and stored in a desiccator until further use. The β-glucan was extracted with a modified protocol proposed by Thammakiti et al. [30]. Briefly, accurately weighed freeze-dried cells were incubated with 30 volumes of 1 M NaOH at 80 °C for 2 h under agitation. After the incubation, samples were centrifuged at 6500×*g* for 10 min. Pellets were washed three times with deionized water and resuspended in equal volumes of 0.5 M acetic acid. Further incubation was performed at 80 °C for 1 h. After the second incubation step, samples were centrifuged at 6500×*g* for 10 min and the resulting pellets were washed 3 times with deionized water. The resulting pellets were freeze-dried and stored in a desiccator until further use.

*Pleurotus citrinopileatus* LGAM 28684 was obtained from the culture collection of the Laboratory of General and Agricultural Microbiology (Agricultural University of Athens). Mycelial biomass was grown in shake flasks, as described in [31]. Briefly, three 1-mm agar plugs from 14-day old mycelium grown in PDA medium, were used as inoculum for 100 mL liquid medium containing xylose 57 g L^−1^, corn steep liquor 37 g L^−1^, K_2_HPO_4_ 1 g L^−1^, and MgSO_4_(H_2_O)_7_ 0.2 g L^−1^ in 250 mL Erlenmeyer flasks. The cultures were incubated for 14 days in a shaking incubator in 28 °C and 100 rpm. Then, the mycelium was removed by filtration, washed and freeze-dried before β-glucan extraction.

Pulsed Electric Field (PEF) treatment of the freeze-dried mycelium was performed in a stainless steel parallel-plate electrode chamber with an electrode gap of 2 cm, with pulses delivered by a pilot scale ELCRACK-5 kW system (DIL, Quakenbrück, Germany). For the treatment, 10 g of freeze-dried mycelium were milled with a mortar and pestle and suspended in 10 mL of deionized water. The sample was allowed to rehydrate for 20 min, was placed in the treatment chamber and 500 near-rectangular bipolar pulses at a pulse width of 15 µs and a frequency of 20 Hz were delivered. The electric field strength achieved was equal to 4.2 kV/cm. After pulsing, the sample was quantitatively recovered from the chamber and brought to a final volume of 100 mL. β-Glucan extraction from PEF-treated and untreated mycelium as control was performed according to the protocol of Wei et al. [32], with some modifications, as previously described [31]. Briefly, the diluted sample was further reflux boiled for 5 days. Deionized water was intermittently added to the apparatus to account for evaporative losses. Solids were removed by centrifugation at 10,000×*g* for 15 min. Two volumes of absolute ethanol were added to the supernatant followed by incubation at 4 °C for 16 h. The solution was centrifuged and the pellet was resuspended in 10 mL deionized water. Finally, β-glucans were precipitated by addition of three volumes of absolute ethanol and subsequent centrifugation.

The content in total glucans and α-glucans were determined in the freeze-dried biomass, according to the mushroom and yeast β-glucan Assay Procedure K-YBGL (Megazyme International Ireland, Bray, Ireland). The β-glucan content was calculated as the difference between total glucans and α-glucans.

### Finizym 250L®-Mediated Transglycosylation of Stevioside

The reactions for Finizym 250L®-mediated transglycosylation of stevioside were set up as follows: 0.01 g β-glucan was solubilized in 50 mM potassium phosphate buffer pH 5. Stevioside was added to a final concentration of 0.5% (w/v), and the reaction was started by the addition of 0.1 mg mL^−1^ Finizym 250L®. The reaction was incubated in a shaking incubator, at 1100 rpm, 60 °C for 24 h, unless otherwise stated. In parallel, proper blank reactions were run, where either thermally inactivated enzyme was added, or β-glucan was omitted in the reaction, in order to correct for possible stevioside hydrolysis. The effect of β-glucan concentration was tested by adding 0.5, 1, 1.5 or 2% (w/v) barley β-glucan to the mixture. The effect of stevioside was tested by adding the acceptor at final concentrations of 0.25, 0.5, 1 or 1.5% (w/v). The effect of enzyme addition was tested by adding 0.26, 0.1, 0.05 or 0.03 mg mL^−1^ enzyme to the reaction.

In the reactions with *S. cerevisiae* β-glucans as sugar donor, the extract was added in the reaction at a final concentration of 3.5% (w/v). Mycelium-extracted β-glucans (with or without PEF treatment) were added at a final concentration of 5.2% (w/v).

### Chromatographic Analysis of Transglycosylation Products

The analysis of the reaction products was performed with a 1260 Infinity HPLC system (Agilent Technologies), equipped with a UV–Vis 1260 Infinity II Variable Wavelength Detector. The sample (20 μL) was loaded on a C18 CC 250/4.6 Nucleosil 100-5 column (Macherey–Nagel), and chromatographic separation was performed with isocratic elution, at 1 mL min^−1^, for 32 min, using as mobile phase 70% (v/v) phosphate buffer pH 2.6 and 30% (v/v) acetonitrile. Monitoring was performed at 210 nm, and stevioside was quantified against a suitable calibration curve.

### Statistical Analysis

Data analysis was performed with SigmaPlot v12.5 software (Systat Software, Inc., San Jose, CA, USA). Error bars represent the standard deviation of the mean value. GraphPad Prism 8 (GraphPad Software San Diego, CA, USA) software was used for statistical analysis (student’s t-test).

## Results and Discussion

### Finizym 250L®-Mediated Transglycosylation of Stevioside with Barley β-Glucan

**Fig. 1:**
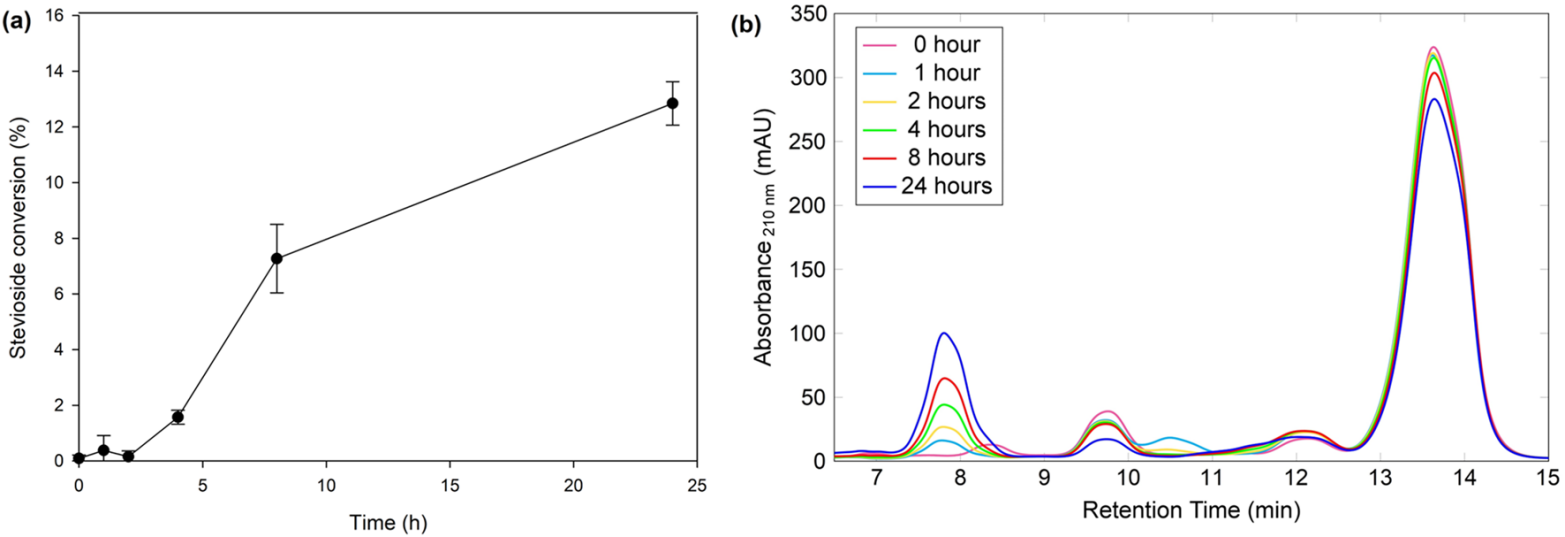
**a** Time course of the Finizym 250L®-mediated transglycosylation reaction, **b** HPLC chromatograms of the different time points of the Finizym 250L®-mediated transglycosylation reaction

The commercial enzyme preparation with β-glucanase activity Finizym 250L® was applied to stevioside conversion towards a more glycosylated product. β-Glucanase Finizym 250L® is of fungal origin, from a *Trichoderma reesei* strain. It was originally designed for the removal of β-glucans from beer mash, to reduce the turbidity of the final product. Therefore, since the enzyme preparation is targeting barley β-glucans, it mainly attacks β-1,3/1,4 glucan polymers. However, since the preparation is derived from the culture supernatant of the fungus, it is possible to also contain β-glucanases with other specificities, and thus to be able to attack β-1,6-linked glucose moieties. The first step was to study the time course of the reaction, and the results are shown in Fig. 1. Up to 24 h, the reduction in stevioside concentration is increasing, reaching 12.9 ± 0.8% stevioside conversion. This is rather unusual for glycosyl hydrolases, since most of them hydrolyze their transglycosylation products in prolonged incubation times [16, 33]. In Fig. 1b, the HPLC chromatograms of the reaction mixtures are shown. The products of stevioside conversion elute earlier from the column, and therefore they appear in shorter retention times. A major product appears in retention time of around 7.8 min, and the corresponding peak is increasing with time. Moreover, a minor product appears in retention time 10.5 min, but the peak disappears in later stages of the reaction, which implies that either this product is further glycosylated and therefore, elutes earlier, or that it is quickly hydrolyzed by the enzyme. Also, the peak corresponding to stevioside (Rt 13.8 min) is decreasing throughout the course of the reaction. Blank reactions without the addition of β-glucan did not show any product formation, and the peak corresponding to stevioside remained unchanged, therefore we concluded that Finizym 250L® is not able to cleave the sugar moieties of stevioside molecule.

β-Glucanases and their nucleophile mutants (glycosynthases) have been used previously for the production of glucosides. β-1,3/1,4-Glucanase from *Bacillus licheniformis* successfully transglycosylated β-fluoride trisaccharides to the corresponding hexasaccharide, at a yield of 20% [34]. Later studies with the nucleophile mutant of the same enzyme achieved a considerably increased yield of 95% [35], and a bi-enzyme system was developed for the production of hexasaccharides from disaccharide starting material [36]. A β-1,3-glucanase from *Cellulomonas cellulans* was also applied to the transglycosylation of 4-nitrophenyl-β-d-glucopyranoside or xylopyranoside, using laminarin as donor, finally achieving a yield of 68% [37].

**Fig. 2:**
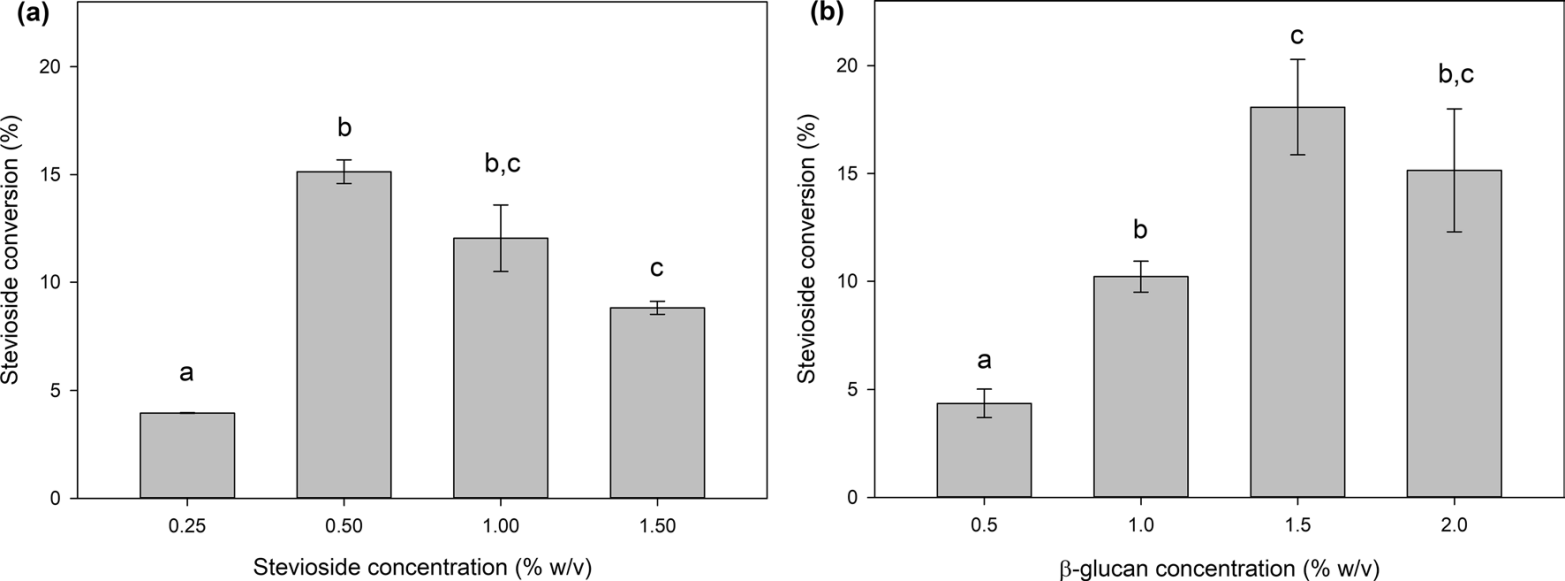
**a** Effect of acceptor concentration (stevioside) on stevioside transglycosylation. **b** Effect of donor concentration (β-glucan) on stevioside transglycosylation. Different letters represent significant differences at p < 0.05 probability level.

Next, the effect of the concentration of acceptor stevioside was studied. The maximum stevioside conversion appeared in 0.5% (w/v) stevioside concentration, and with increasing stevioside concentrations the conversion was decreasing, although the differences observed among the different stevioside concentrations were not significant (Fig. 2a). Stevioside conversion reached up to 15.1 ± 0.6% in this case. However, calculating the mM of transglycosylated stevioside in each case, based on a stoichiometric conversion, the product concentration increased with increasing stevioside concentrations, up to 1.7 mM product in the highest concentration tested.

In order to increase the production yields, the effect of β-glucan concentration was also tested. As shown in Fig. 2b, increasing the concentration of the donor to 1.5% (w/v) led to an increase in stevioside conversion up to 18.1 ± 2.2%, but further increase in β-glucan concentration did not lead to higher conversion yields.

**Fig. 3:**
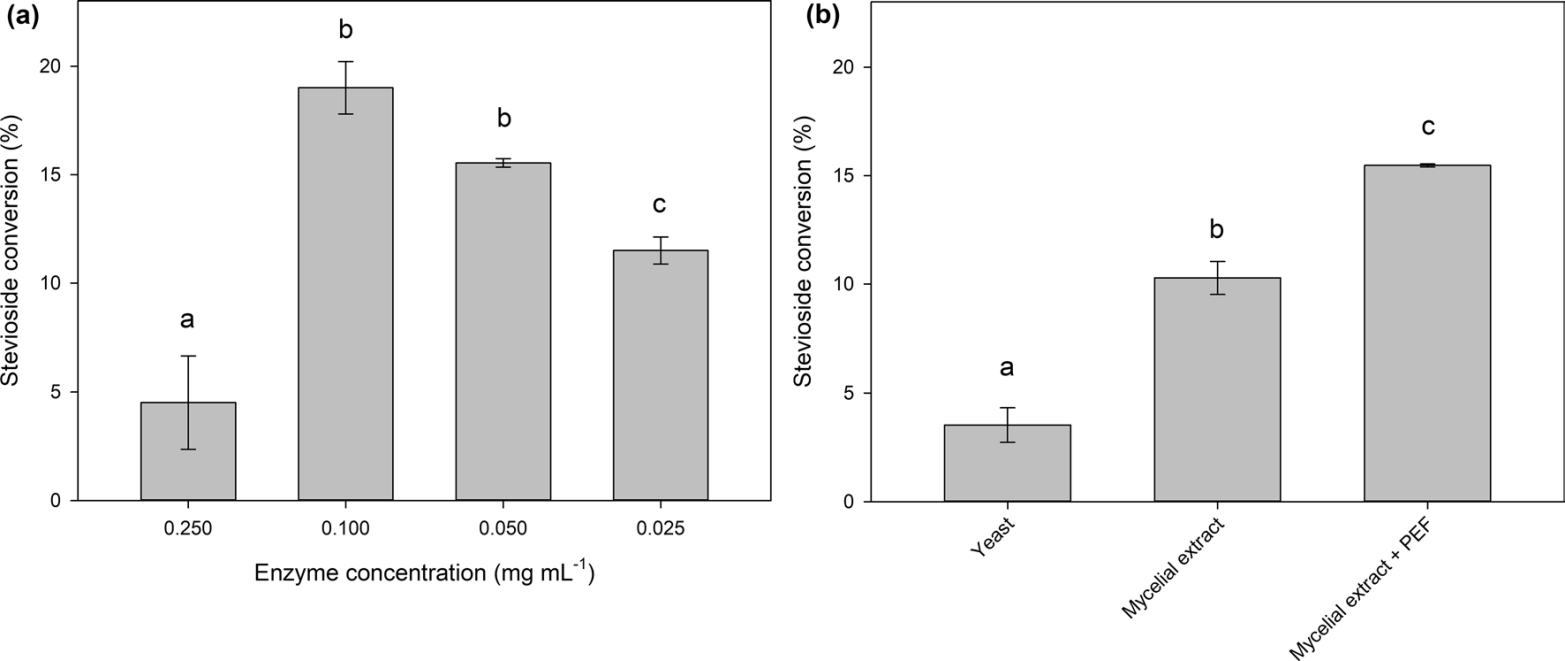
**a** Effect of enzyme concentration on stevioside transglycosylation, **b** Finizym 250L®-mediated transglycosylation of stevioside, with different microbial biomass extracts as β-glucan donors. Different letters represent significant differences at p < 0.05 probability level

The effect of enzyme concentration was also tested, in reactions with the optimum conditions from the previous experiments, and the results are shown in Fig. 3a. The optimum enzyme concentration was found to be 0.1 mg mL^−1^, reaching up to 19.0 ± 1.2% stevioside conversion, although the difference between 0.1 and 0.05 mg mL^−1^ was not statistically significant.

The obtained conversion yields were similar to those obtained from other glucosidases from our previous work [16, 33]. Generally, the transglycosylation yields obtained from glucosidases are low, from 15 to 30%, and modern approaches include protein engineering and directed evolution in order to obtain mutant enzymes with minimal hydrolytic activity [38, 39]. Nonetheless, it is of high importance to discover and characterize new enzymes with the ability to catalyse specific transglycosylations, as potential targets for the development of evolved variants. Specifically, for stevioside transglycosylation, the addition of β-linked glucosyl moieties to the molecule, could result to next-generation sweeteners with improved properties, and, most importantly, with minimal caloric value. β-linked glycosidic bonds are not hydrolysed in the human body (with an exception of β-linked galactose in the lactose molecule, catalysed by human β-galactosidase in the majority of adults), and therefore, the insertion of β-linked glucose to the stevioside molecule retains the caloric value of the product, and concomitantly improves the sensory characteristics, as described in previous studies [2, 7, 16, 17]. Therefore, it is of high importance to uncover the potential of already commercially available biocatalysts in inserting β-bonds to the desired products. Taking into consideration the anti-inflammatory and prebiotic activities of natural steviol glucosides [40, 41], biocatalysis could offer the possibility to construct libraries of similar compounds, to screen for improved bioactivities.

### Fungal β-Glucans as Sugar Donors for Stevioside Transglycosylation

In the above experimental setup, commercially available β-glucan from barley was used as sugar donor for the transglycosylation reaction. However, the isolation of barley β-glucan is a costly and time-consuming process. Moreover, barley β-glucan is used as a supplement in many food products for human consumption, due to its beneficial effects in the human health, such as anti-diabetic, and cardioprotective properties [42]. In the context of circular bioeconomy and biorefinery, the use of alternative sugar donors was tested for the Finizym 250L®-mediated transglycosylation of stevioside, aiming at the valorization of β-glucan from a low-cost source, non-competitive with human nutrition, preferably a side-product from other bioprocesses. Due to the fungal origin of the enzyme, fungal β-glucan sources were chosen. *S. cerevisiae* biomass is produced as a side product in many food and bioproduct processes, such as brewing or yeast extract production, and is a valuable resource of β-glucan. In the relevant literature, many protocols have been described for the isolation of β-glucans from yeast biomass. PEFs are commonly applied for the extraction of intracellular compounds, since they can increase the cell membrane and cell wall permeability. The effects of PEF as a pretreatment to the extraction of cell wall material has not been reported. Nevertheless, there is strong evidence that the exposure to the electric field significantly increases the porosity of polysaccharide-rich fungal cell walls [43, 44]. This effect may contribute indirectly to the effectiveness of extraction processes that rely on the diffusion of solvents, acids, bases or enzymes through the dense polysaccharide matrix.

The mycelial biomass of *Pleurotus* was also extracted, aiming at the isolation of β-glucan constituents of the cell wall. The isolation protocol performed was described in our previous work [31], and the effect of PEF treatment was also examined, regarding the production of an extract which can be efficiently used as sugar donor for the Finizym 250L®-mediated transglycosylation of stevioside. The results of the β-glucan content for the obtained extracts are shown in Table 1. To the best of our knowledge, this is the first time that the PEF method is applied to the isolation of β-glucans from the mycelium of a white-rot fungus, although a galactan was previously extracted from *Morchella esculenta* with a PEF-assisted extraction method [45].

**Table 1:** Total glucan, α- and β-glucan content of the fungal extracts used as donors for the Finizym 250L®-mediated transglycosylation of stevioside

Extract	Total glucan (% w/w)	α-Glucan (% w/w)	β-Glucan (% w/w)
*S. cerevisiae*	63.2 ± 0.3	3.4 ± 0.2	59.8 ± 0.7
Supernatant from *P. citrinopileatus* biomass extract	51.8 ± 0.4	20.7 ± 0.6	31.2 ± 0.2
Supernatant from PEF-treated *P. citrinopileatus* biomass extract	53.5 ± 1.7	15.8 ± 1.3	37.7 ± 0.4
Solid residue from *P. citrinopileatus* biomass extract	8.0 ± 0.4	1.4 ± 0.1	6.6 ± 0.4
Solid residue from PEF-treated *P. citrinopileatus* biomass extract	7.7 ± 0.8	1.5 ± 0.1	6.2 ± 0.8

As shown in Table 1, the PEF-treated extracts were enriched in β-glucans, compared to the untreated controls, but also the amount of α-glucan content was significantly lower, ultimately achieving a more β-glucan-enriched material. The extracts were applied as sugar donors for the Finizym 250L®-mediated transglycosylation of stevioside, and the results are shown in Fig. 3b. Statistically significant differences were observed among all the different β-glucan sources tested, indicating that this factor significantly affects the transglycosylation of stevioside. The most effective sugar donor was found to be the PEF-extracted *Pleurotus* biomass, reaching 15.5% stevioside conversion, which is quite similar to the yields obtained with the commercially available barley β-glucan, before the optimization steps. This holds great potential for the valorization of spent fungal biomass, especially for applications where white-rot fungi are used, such as bioremediation applications, or the production of edible basidiocarps.

β-Glucans from *Pleurotus* species have been extensively studied, due to their immunomodulatory, prebiotic and anticancer activities [46–49]. Regarding *P. citrinopileatus* in particular, only one type of β-glucan has been isolated and described, a polymer consisting of β-1,3-linked β-d-glucopyranosyl residues, substituted with β-1,6-linked side chain glucopyranosyl residues [50], which was later shown to have a potent immunomodulatory activity, activating human dendritic cells [51]. This β-1,6-branched β-1,3-glucan is common in various *Pleurotus* species, such as *P. albidus* [52], *P. eryngii* [53], *P. ostreatus* [54], *P. sajor-caju* [47], among others. Nonetheless, many more β-glucans have been described from other *Pleurotus* mushrooms, such as linear β-1,3-glucans [46, 48] and linear β-1,6-glucans [55], but also β-1,3/1,6-glucans with α-linked-1,6 substitutions [56].

β-Glucans from *S. cerevisiae* present similar heterogeneity. The predominant β-glucan in the cell walls of *S. cerevisiae* is the branched β-1,3/1,6-polymer [57, 58], while linear β-1,3 and β-1,6 glucans have also been described [59–62]. Obviously, it is unclear why the fungal β-glucanase performed better with the mushroom β-glucan extract as glycosyl donor, compared to β-glucan from yeast. It is possible that the superiority of the mushroom extracts is due to their higher solubility in the water-based reaction systems used in this study, in contrast with the β-glucans from the yeast, which are mainly soluble in basic pH. However, it is also possible that some aspects of the β-glucan architecture from fungi are still elusive, and perhaps there are more differences in the chemistry of the β-glucans from various sources than what we know so far.

Taking all of the above into consideration, the process described in this work could be an object to further optimization steps, including protein engineering. Nonetheless, the utilization of spent fungal biomass from other bioprocesses could significantly contribute to the sustainability and the cost effectiveness of the process. Moreover, the residual β-glucan remaining after reaction completion is still a valuable bioproduct, due to its multiple beneficial effects on human health [63, 64]. Pleuran in particular, a β-glucan from *Pleurotus* mushrooms, is found to relieve respiratory tract infections in athletes [65] and children [66], and may even be used for the treatment of COVID-19 symptoms [67]. Pleuran is also examined as an active ingredient for atopic dermatitis treatment [68], while it is also tested in clinical trials for gastroenteritis treatment [69]. Therefore, depending on the desired application, the reaction mixture might even be used without further purification steps, aside from enzyme removal.

## Conclusions

In the present work, an enzymatic process for the modification of stevioside is described. The commercial β-glucanase Finizym 250L® was employed for the transglycosylation of stevioside, with the aim to produce alternative sweeteners with potentially improved properties. For the first time in this study, barley β-glucan, as well as fungal β-glucans from spent biomass were used as alternative sugar donors, resulting in similar conversion yields. The proposed system could be used for the sustainable upgrading of stevia-based sweeteners, with the simultaneous valorization of byproducts from other food-related applications, towards the design of modern biorefinery processes, which could be further implemented on site, in the same production plant. Fungal spent biomass in particular, despite its numerous health benefits, is a rather untapped source for the production of nutraceuticals and functional foods. Nonetheless, the development of modern extraction methods for the isolation of β-glucans could provide a valuable tool for the commercialization of such materials, and their incorporation in the design of next-generation functional food products.

## Data Availability

Data will be available on reasonable request.
